# Partnership and marriage and risk of type 2 diabetes: a narrative review

**DOI:** 10.1007/s00125-025-06360-3

**Published:** 2025-02-07

**Authors:** Bernd Kowall, Wolfgang Rathmann

**Affiliations:** 1 Institute for Medical Informatics, Biometry and Epidemiology, University Hospital Essen, Essen, Germany; 2 Institute for Biometrics and Epidemiology, German Diabetes Center, Leibniz Center for Diabetes Research at Heinrich Heine University Düsseldorf, Düsseldorf, Germany

**Keywords:** Couple-based intervention, Marriage, Partnership, Review, Spousal concordance, Spouse, Type 2 diabetes

## Abstract

In this review, we discuss how partnership and marriage influence cardiometabolic risk factors and risk of type 2 diabetes, and how couple-based approaches to type 2 diabetes prevention might complement individual-focused prevention efforts. There is some evidence that being married per se has a small positive effect on type 2 diabetes risk. Moreover, there is spousal concordance for many type 2 diabetes risk factors due to assortative mating and convergence during partnership, with weak to moderate correlations found for anthropometric measures, blood pressure, lipid concentrations, smoking, alcohol consumption and physical activity level. A meta-analysis shows that people have a higher risk of type 2 diabetes if their spouse has diabetes (OR 1.72, 95% CI 1.47, 2.02). However, despite some evidence, there is still a lack of research on similar associations in relation to progression to type 2 diabetes and diabetes complications. Several studies have suggested that behaviour changes, for example smoking cessation or weight loss, in one partner increase the likelihood that the other partner will make the same changes. Subsequent studies of couple-based interventions that focus on both partners have shown that people are more likely to adhere to a diabetes prevention programme if their partners are also involved in the programme. However, the effect of the quality of marriage on the outcome of an intervention is still unclear. Couple-based interventions are promising, but there is a lack of RCTs comparing couple-based interventions with individual-centred interventions.

**Graphical Abstract:**

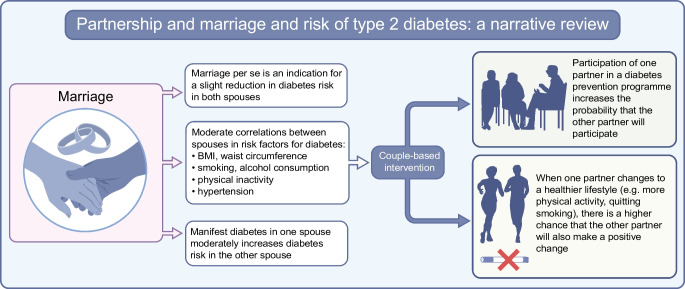

**Supplementary Information:**

The online version of this article (10.1007/s00125-025-06360-3) contains a slide of the figure for download.

## Introduction

Type 2 diabetes prevention is currently mainly focused on individuals, who are advised to eat less and more healthily, be more physically active and stop harmful behaviours such as smoking and excessive alcohol consumption. This requires a lot of willpower on the part of the individual and is not always easy to maintain [1]. The risk of developing type 2 diabetes and the self-management of existing diabetes also depend on factors in the social environment, which range from partnerships, families and social networks to neighbourhoods and features of the built environment, societal norms and legal regulations [1–3].

**Fig. 1:**
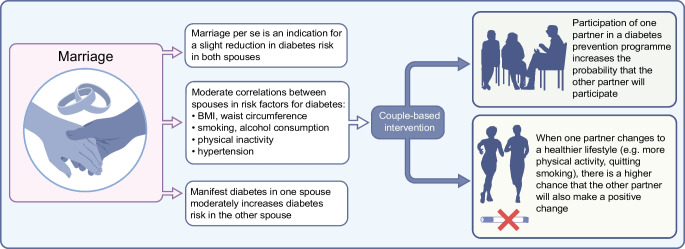
Marriage, risk of diabetes and benefits of couple-based intervention. This figure is available as a downloadable slide

This narrative review focuses on the importance of partnership and marriage for the development and progression of type 2 diabetes. First, we discuss how marriage itself influences glycaemic outcomes. Second, we summarise which risk factors are responsible for spousal concordance in type 2 diabetes. Third, we look at the effect of type 2 diabetes in one spouse on the risk of diabetes in the other spouse. Finally, we address how these findings can be used for couple-based type 2 diabetes prevention. An overview of this review is provided in Fig. 1.

In this narrative review we relied on meta-analyses where possible. We report the results of meta-analyses for associations between living alone and type 2 diabetes, spousal correlations for type 2 diabetes risk factors and spousal concordance for type 2 diabetes. However, for the association between marriage per se and type 2 diabetes, we report individual studies because there are few publications on this topic and no meta-analyses have been published to date. For the concordance of type 2 diabetes in spouses, we added recently published individual studies that were not included in the previous meta-analysis from 2019. For couple-based interventions aimed at preventing type 2 diabetes, no meta-analyses are available because of the small number of published studies on this topic and their heterogeneity. The studies included in this review generally did not explicitly mention same-sex couples; the data included are therefore mainly from opposite-sex couples.

## Association of marriage per se with type 2 diabetes

When considering the association between marriage and type 2 diabetes it should be noted that there is a selection effect in marriage; that is, chronically sick people generally have a lower chance of finding a spouse [4]. The few prospective studies on the association between marriage per se and incident type 2 diabetes indicate a possible prospective effect of marriage. One study with 22 years of follow-up found a slightly increased risk of type 2 diabetes in unmarried compared with married men (RR 1.16, 95% CI 1.04, 1.30) [5]. Another study in people with abdominal obesity found an increased type 2 diabetes risk in single and divorced or widowed people; however, the estimates were rather imprecise (OR 1.46 [95% CI 0.49, 4.39] and OR 1.32 [95% CI 0.58, 3.00], respectively; reference: married people) [6]. In contrast, in a study from Brazil, the proportions of people with incident type 2 diabetes were similar among those who were married and those who were single [7]. Interestingly, the English Longitudinal Study of Ageing (ELSA) found that married/cohabitating people without previously diagnosed type 2 diabetes had lower HbA_1c_ levels than those without a partner; after adjustment for sociodemographic factors, lifestyle, social support, depression, BMI and weight, the difference in HbA_1c_ was –2.3 mmol/mol (95% CI –3.4 mmol/mol, –1.1 mmol/mol) (−0.21% [95% CI −0.31%, −0.10%]) [8]. The odds of developing prediabetes (HbA_1c_ ≥39 mmol/mol and <48 mmol/mol [≥5.7% and <6.5%]) was also lower in married/cohabitating couples than in those without a partner (OR 0.43 [95% CI 0.20, 0.93]), but because of the small number of incident diabetes cases the OR for type 2 diabetes could not be precisely estimated (OR 0.68 [95% CI 0.13, 3.55]) [8]. Effect estimates for the association between marital status and type 2 diabetes may be biased by inadequate adjustment for confounding. Assuming that less healthy people are more often unmarried, health characteristics in younger years of life may be related to marital status and also to health in later years. Therefore, general health may be a confounder that is often missed in analyses. However, anthropometric measures, hypertension, lipid levels and depression in later life may be a result of marital status and, therefore, mediators. Adjusting for mediators may lead to an underestimate of effects.

According to several cross-sectional studies and a meta-analysis of longitudinal studies, living alone is associated with an increased risk of type 2 diabetes [9–12]. The meta-analysis included eight cohort studies (six from Western Europe, two from Eastern Asia) with follow-up times between 0.25 and 14 years, HRs ranging from 0.94 to 1.97 and moderate to substantial heterogeneity (*I*^2^=0.60) [12]. The meta-analysis showed a slightly increased risk of type 2 diabetes in those living alone compared with those living with others (HR 1.24 [95% CI 1.06, 1.46]), which was more pronounced in men than in women [12]. One of the proposed mechanisms for this association is that people living alone have higher stress levels because living with others buffers stress, which is an established risk factor for type 2 diabetes [12, 13]. Furthermore, people living alone tend to have a less healthy diet, for example eat less vegetables, fruit and fish, which is linked to chronic diseases such as diabetes [14]. Finally, it is plausible that lack of a close confidant and lack of social support may reduce knowledge of health-related information, including information on diabetes prevention [13].

## Spousal concordance in cardiometabolic risk factors

Two meta-analyses provide strong evidence of spousal concordance in shared cardiometabolic risk factors [15, 16]. The first was based on 207 cohorts and data from more than 100,000 couples. Separate meta-analyses were performed for systolic blood pressure (based on 30 studies), diastolic blood pressure (27 studies), lipid and glucose concentrations (seven to 23 studies), anthropometric measures (four to 19 studies) and smoking (seven studies) [15]. The correlation coefficients were all positive but small, ranging from 0.07 for total cholesterol to 0.10 for waist circumference, 0.15 for BMI and 0.23 for smoking. This means that the percentage of variance explained ranged from 0.5% for total cholesterol to 5.3% for smoking. The second meta-analysis of 22 traits was based on 480 partner correlations from 199 studies [16]. In this meta-analysis, *I*^2^ for the different traits ranged from 0.54 for general anxiety to 0.99 for educational attainment, indicating substantial heterogeneity. However, the authors emphasised that the high *I*^2^ values may be due to the small within-study variance and that the median of tau as an alternative measure of heterogeneity was 0.09, indicating a lower level of between-study variance. The highest correlation coefficients were found for political values (0.58), religiosity (0.56), educational level (0.55) and intelligence quotient (0.44). For anthropometric measures, correlation coefficients ranged from 0.16 for BMI to 0.24 for height. Moderate correlations were found for alcohol consumption (0.42) and smoking quantity (0.29) [16]. Interestingly, the correlation for quitting smoking was 0.54, which was higher than that for smoking. This suggests that behavioural change in one partner may be a model for behavioural change in the other partner. Furthermore, the Maastricht Study found that spousal concordance in risk factors for type 2 diabetes was weaker the further downwards in the causal cascade a risk factor was located [17]. For men, the multivariable-adjusted Spearman correlation coefficients were 0.42 for the Dutch Healthy Diet index, 0.35 for time spent performing high-intensity physical activity, 0.28 for percentage body fat and 0.15 for HbA_1c_ level; the smallest correlation coefficients were found for markers of glucose metabolism, such as the Matsuda Index and the disposition index (0.00 and 0.09, respectively). For the concordance of hypertension in married couples, a meta-analysis of seven cross-sectional studies and one case–control study showed a 41% higher odds of hypertension in individuals whose spouse had hypertension (OR 1.41 [95% CI 1.21, 1.64]) [18].

Assortative mating and convergence are possible explanations for spousal concordance in cardiometabolic risk factors [15, 19, 20]. Assortative mating means that people prefer partners with similar characteristics, such as education level, attitudes and behaviours. Convergence over time can occur from sharing the same environment, the same financial resources and the same social network [19]. It can also result from social control, for example one partner controlling the other’s health behaviour [19]. To distinguish between assortative mating and convergence, longitudinal analyses are necessary to observe changes over time. Several studies have suggested assortative mating as the underlying cause of spousal concordance in cardiovascular risk factors. In the meta-analysis by Di Castelnuovo et al, among the included studies with longitudinal data, the proportion of correlations that increased over time was generally small, for example reported in three of 18 longitudinal studies for systolic blood pressure and one of five longitudinal studies for BMI [15]. In a further study, correlations in cardiovascular risk factors (systolic and diastolic blood pressure, BMI, forced expiratory volume) decreased with marriage duration, which is more likely to be explained by assortative mating than partners sharing the same environment [21]. In a further study from China, moderate spousal concordance in cardiometabolic risk factors was observed in young, newly married couples, supporting assortative mating as a possible explanation [22]. Several studies have also suggested that assortative mating has a major influence on spousal concordance in smoking [15, 20].

Behaviour change may contribute to the convergence of lifestyles in couples. Several studies have shown that a change in health behaviour in one partner increases the likelihood that the other partner will make the same change [23, 24]. In one study, participants were more likely to quit smoking if their spouse had quit smoking than if their spouse had always been a non-smoker. The same was true for becoming more physically active or losing weight [23]. This influence of one partner’s behavioural change on the other partner’s behaviour was strongest for smoking, but also considerable for physical activity and weight loss [23]. In the Atherosclerosis Risk in Communities Study (ARIC), HRs were estimated for husbands’ incident obesity depending on their wives’ obesity status [24]. If wives without obesity developed obesity, the HR for incident obesity of their husbands was 1.8 (95% CI 1.3, 2.4); if wives with obesity became non-obese or remained obese, the HRs were 1.2 (95% CI 0.7, 2.0) and 1.1 (95% CI 0.9, 1.3), respectively (reference: wife without obesity with stable weight) [24]. This demonstrates that spouses may benefit from weight stabilisation or weight loss in their partners.

## Diabetes in one spouse and risk of diabetes in the other

Two previous meta-analyses, including three and five cross-sectional studies, respectively, evaluated whether individuals have a higher risk of diabetes if their spouse has diabetes [15, 25]. Both studies showed a small increase in diabetes risk when a spouse has diabetes (OR 1.16 [95% CI 1.02, 1.31] and 1.18 [0.97, 1.40], respectively). A third meta-analysis, published in 2019 and including 17 studies (nine cross-sectional, five cohort and three case–control studies) [26], found a considerably larger positive association between spousal type 2 diabetes status and the development of diabetes (pooled OR 1.88 [95% CI 1.52, 2.33]). *I*^2^ was 0.98 for the overall estimate, indicating strong heterogeneity (i.e. that 98% of the variability between the studies was due to heterogeneity and not to chance). After exclusion of seven studies whose effect estimates were considered outliers, *I*^2^ was reduced to 0.18 and the pooled OR was 1.33 (95% CI 1.24, 1.42). Thus, the pooled effect was strongly reduced, but still indicated an increased odds of diabetes in partners of individuals with diabetes [26]. The high OR for all 17 studies was largely due to the inclusion of a study by Cunningham et al (*N*=180,930), which showed very strong effects (OR 8.7 [95% CI 7.4, 10.2]) [27]. A smaller study by Mothojakan et al with 201 participants showed an OR of 7.2 (95% CI 2.9, 18.0) [28]. Excluding the study by Cunningham gave a pooled OR of 1.61 (95% CI 1.41, 1.84) [26].

Two questions about spousal diabetes as a risk factor for diabetes are raised by these studies: first, what effect including subsequently published studies not yet included in the third meta-analysis [26] would have and, second, what kind of confounding adjustment is appropriate to estimate the effect of spousal diabetes on a partner’s diabetes status. Of the studies published to date, some made no adjustments, whereas others adjusted for a large set of potential confounders.

Two cohort and four cross-sectional studies on spousal diabetes status and the development of diabetes in the other spouse have been published since the 2019 meta-analysis; in addition, another small cross-sectional study published in 2019 was restricted to couples with wives who had a history of gestational diabetes [29–35]. These studies are summarised in Table 1. When all these seven recent studies were additionally included, the pooled OR for the association between spousal diabetes status and the development of diabetes in the other spouse was slightly lower (OR 1.72 [95% CI 1.47, 2.02]). When the study by Cunningham et al [27] was omitted, addition of the new studies resulted in an even lower pooled OR of 1.54 (95% CI 1.33, 1.71).

**Table 1:** Characteristics of studies not included in the meta-analysis by Appiah et al [26] that assessed the association of spousal diabetes status with the development of diabetes in a partner

Study (first author, year, country)	Type of study	Association	Unadjusted effect measure (95% CI)	Adjusted effect measure (95% CI)	Largest adjustment set
Goyal (2019) India [29]	Cross-sectional; inclusion of couples with wives with a history of gestational diabetes	Exposure: FPG ≥7.0 mmol/l or 2hPG ≥11.1 mmol/l or HbA_1c_ ≥48 mmol/mol (≥6.5%) in one partner Outcome: diabetes according to the same criteria in the other partner	Not reported	OR 2.60 (0.78, 8.67)	Age, family history of diabetes, education, occupation, marriage duration, family structure, partner’s metabolic variables (dysglycaemia, overweight/obesity) Wives: additional adjustment for insulin use during pregnancy
Ramezankhani (2019) Iran [30]	Cohort study; follow-up ≥15 years	Exposure: FPG ≥7.0 mmol/l or 2hPG ≥11.1 mmol/l or intake of glucose-lowering drugs in one spouse Outcome: diabetes according to the same criteria in the other spouse	Not reported	HR 1.38 (1.03, 1.84) (exposure: diabetes in husbands; outcome: diabetes in wives) HR 1.03 (0.72, 1.48) (exposure: diabetes in wives; outcome: diabetes in husbands) Pooled HR 1.22 (0.92, 1.62)	Age, SES, family history of diabetes, physical activity
Jun (2020) Korea [31]	Cross-sectional	Exposure: self-report of diabetes, or intake of glucose-lowering drugs/insulin stated in medical records Outcome: diabetes according to the same criteria in the other spouse	Not reported	OR 1.64 (0.92, 2.92) (exposure: diabetes in husbands; outcome: diabetes in wives) OR 1.70 (0.96, 3.00) (exposure: diabetes in wives; outcome: diabetes in husbands) Pooled OR 1.67 (1.11, 2.51)	Age, education, income
Watanabe (2020) Japan [32]	Cross-sectional	Exposure: husbands undergoing diabetes therapy Outcome: wives undergoing diabetes therapy	Not reported	OR 1.45 (1.34, 1.58) (exposure: diabetes in husbands; outcome: diabetes in wives) OR 1.44 (1.32, 1.56) (exposure: diabetes in wives; outcome: diabetes in husbands) Pooled OR 1.45 (1.36, 1.53)	Wife’s age, household expenditure, location of residence, education, smoking, alcohol consumption, therapy for another disease and its interaction with age group
Nielsen (2023) India and Pakistan [33]	Cross-sectional	Exposure: prior diagnosis of diabetes, or intake of glucose-lowering drugs or FPG ≥7.0 mmol/l or HbA_1c_ ≥48 mmol/mol (≥6.5%) in one spouse Outcome: diabetes according to the same condition in the other spouse	Not reported	OR 1.76 (1.43, 2.18) (exposure: diabetes in husbands; outcome: diabetes in wives) OR 1.78 (1.44, 2.20) (exposure: diabetes in wives; outcome: diabetes in husbands) Pooled OR 1.77 (1.52, 2.06)	Use of inverse probability weighting (age, education, sex, self-reported diabetes status, smoking, alcohol consumption, sedentary behaviour, vegetarian diet, education, occupation, SES tertile)
Zhao (2023) China [34]	Cohort study; mean follow-up 3.6 years	Exposure: diabetes (FPG ≥7.0 mmol/l or 2hPG ≥11.1 mmol/l or HbA_1c_ ≥48 mmol/mol [≥6.5%]) in one spouse Outcome: incident diabetes according to the same criteria in the other spouse	HR 1.25 (1.11, 1.40)	HR 1.15 (1.03, 1.30) Effect modification by diabetes control: HR 1.10 (0.92, 1.30) when spouse has diabetes with HbA_1c_ <53 mmol/mol (7.0%); HR 1.20 (1.04, 1.39) when spouse has diabetes with HbA_1c_ ≥53 mmol/mol (7.0%)	Age, sex, education, family history of diabetes, income, urban residence, obesity, hypertension, dyslipidaemia, prediabetes status, diet score, physical activity, sleep, smoking, ICVHM factors
Brieger (2024) Germany [35]	Cross-sectional	Exposure: self-report of diabetes, intake of glucose-lowering drugs or insulin, or HbA_1c_ ≥48 mmol/mol (≥6.5%) in one spouse Outcome: diabetes according to the same criteria in the other spouse	OR 1.17 (0.83, 1.66)	OR 1.07 (0.74, 1.54)	Age, education, alcohol consumption, dietary pattern index, smoking, physical activity, BMI, cholesterol and triglyceride levels

2hPG, 2 h plasma glucose; FPG, fasting plasma glucose; ICVHM, ideal cardiovascular health metrics; SES, socioeconomic status

In several studies, the effect of husbands’ diabetes on risk of diabetes in their wives was compared with the effect of wives’ diabetes on risk of diabetes in their husbands. The results were inconclusive: in two studies, the effect of husbands’ diabetes was greater than the effect of wives’ diabetes [30, 36], in one study the effect of wives’ diabetes was greater than the effect of husbands’ diabetes [37] and in three studies there was virtually no difference in effect [31–33]. An interesting finding, which needs to be confirmed in further studies, was that the association between diabetes in one spouse and the risk of diabetes in the other was stronger when the first spouse’s diabetes was poorly managed [34].

There are only a few causal chains leading from diabetes in one partner to diabetes in the other. It is possible that having a partner with diabetes makes the other partner aware of the risk of developing the disease and leads to a change in lifestyle. It is also plausible that the partner with diabetes changes their lifestyle, for example by eating more healthily, and that the other partner participates in this lifestyle change. However, both of these mechanisms would result in diabetes in one partner reducing the risk of diabetes in the other. It is plausible that there is a causal chain mediated by stress: diabetes in one partner may cause distress in the other partner, which in turn increases the risk of diabetes in the partner. There is some evidence that stress is a risk factor for diabetes [38, 39]; however, evidence for the first part of the causal chain is limited. One study found high levels of distress in partners of individuals with type 1 diabetes; in particular, 64% of partners reported distress related to hypoglycaemia [40, 41]. Furthermore, in the German sample of the international DAWN2 (Diabetes, Attitudes, Wishes and Needs) study, family members experienced similar diabetes-related stress as relatives with diabetes [42]. Finally, in a diary study of 199 romantic partners of individuals with diabetes, 52% of partners reported ever experiencing diabetes-related stress, often associated with low or high blood glucose levels [43].

As causal mechanisms linking diabetes in one partner to a higher risk of diabetes in the other are rare, the observed associations could mainly be due to confounding. When published effect estimates are >1, this may reflect, for example, mediation by stress, but is more likely to reflect the presence of residual confounding. The latter occurs because, for example, lifestyle factors, such as diet or physical activity, are often not measured accurately. In addition, other potential confounders that may be overlooked include characteristics of the microbiome, which are similar in spouses and have direct and indirect effects on glucose levels, and characteristics of the built environment [2, 44, 45].

In addition, marital discord has been proposed to explain how the risk of diabetes is increased in both partners, through two separate pathways [46]. First, marital discord is a strong risk factor for depression, which promotes inflammation and in turn is associated with diabetes. Second, people in strained marriages are more likely to have sleep problems, which is a well-established risk factor for diabetes [47, 48].

However, when considering whether lifestyle interventions or screening should be targeted at married couples, it is not the true causal effect that is of interest, but the mere association. What a general practitioner wants to know is how much the risk of diabetes is increased for a person whose partner has diabetes, regardless of the basis of the association. Therefore, if the aim of the studies on spousal concordance is purely associational, there is no need to adjust for confounding [49]. Crude effect estimates are therefore particularly important for the question of couple-based interventions, although they were not reported in many of the studies identified in this review. Adjusted regression models are useful to gain insight into which common risk factors mainly contribute to the joint risk of diabetes in married couples.

There is still little evidence available on the relationship between diabetes in one spouse and prediabetes, progression of diabetes and diabetes complications in the other spouse. In a cross-sectional analysis from the German Heinz Nixdorf Recall Study, diabetes in one spouse was associated with a higher chance of diabetes or prediabetes (≥39 mmol/mol and <48 mmol/mol [≥5.7% and <6.5%]) in the other spouse (crude OR 1.20 [95% CI 1.06, 1.35]), adjusted OR 1.12 [95% CI 0.99, 1.28]) [35]. In a cross-sectional study from Taiwan, the probability of having prediabetes (i.e. HbA_1c_ 39–46 mmol/mol [5.7–6.4%]) was higher for relatives and spouses of individuals with diabetes than for unrelated control participants (54%, 55% and 35%, respectively). However, this study was based on a small sample size of only 37 spouses, 98 relatives and 40 control participants [50].

Finally, there are only a few studies available on the association between marriage or partnership and risk of diabetes complications in people with diabetes. A cross-sectional study from China showed that people with diabetes had a lower chance of developing retinopathy if their spouse also had diabetes (adjusted OR 0.64 [95% CI 0.41, 0.98]) [51]. The authors explained this result by the fact that joint management is more successful in controlling diabetes than when an individual manages the disease alone. Furthermore, in postmenopausal women with type 2 diabetes from the Women’s Health Initiative, those who were married or who lived in an intimate partnership had a lower risk of coronary heart disease (adjusted HR 0.82 [95% CI 0.69, 0.97]) [52]. It is conceivable that spouses and partners help individuals with diabetes maintain their daily management routines and cope better with diabetes distress.

## Couple-based type 2 diabetes prevention

A high concordance of health behaviour changes forms the empirical basis for couple-based interventions in type 2 diabetes prevention [23]. For example, when one partner changes to a healthier lifestyle (e.g. quitting smoking, becoming more physically active), there is a higher chance that the other partner will also make a positive change [23].

However, a definition of and framework for couple-based type 2 diabetes prevention needs to be established. Interventions could include educating one partner as a coach, who then assists the other partner with an increased diabetes risk to make positive health behaviour changes [53]. A second approach could be to focus on both partners and communication around health behaviours [53]. It is noteworthy that these interventions should not provide information developed for individuals, but rather introduce strategies for couples to support each other.

Five main domains need to be addressed in interventions for couple-based type 2 diabetes prevention: (1) education about type 2 diabetes and its main risk factors; (2) sharing the couple’s thoughts and feelings regarding type 2 diabetes; (3) making decisions on preventive actions; (4) implementing changes that result from the interventions; and (5) addressing partnership functioning unrelated to type 2 diabetes [53].

An important prerequisite is that couples are willing to openly communicate [54]. Therefore, couple-based interventions may not be appropriate if there has been frequent criticism and low support in a relationship, or partner violence [54]. On the other hand, couple-based interventions may increase support within the marriage, even in difficult relationships. Social support is the main source of self-efficacy, which enhances and maintains changes in health behaviour [55]. Self-efficacy is the belief in one’s own capacity to reach specific goals. In the older population, which is the main target group for diabetes prevention, spouses are becoming the main source of social support, but only if marital satisfaction is high [56].

### Evidence for couple-based type 2 diabetes prevention

Currently, only a few studies have evaluated couple-based type 2 diabetes prevention, despite the rising prevalence of obesity and type 2 diabetes. The role of including partners in the National Diabetes Prevention Program (NDPP) in the USA has been evaluated in an observational study comprising 62% Hispanic participants and 19% African American participants; most enrollees were women (79%) [54]. Participants with a shared address were considered as dyads. NDPP delivery was identical whether participants enrolled with a household member or alone. Interestingly, programme participation was higher in dyad members, with a threefold increased chance of attending more than one programme session compared with people enrolling alone [54]. Furthermore, participants with an additional household member had a twofold higher chance of completing all NDPP sessions [54]. There was no overall effect of dyad status on the success of weight loss. However, there was an interaction with sex with respect to change in body weight. Men participating with female peers had a fourfold increased odds of achieving ≥5% weight reduction compared with men participating without a partner [54]. No difference in weight change was found in women between those participating with a partner and those participating without. Thus, men may have benefited from participating female partners, who may take more responsibility for facilitating household changes (e.g. in nutrition).

In line with these findings, in the Special Diabetes Prevention Program for Indians, participants who had no family support person had higher short-term and long-term risks of not completing all sessions [57]. Thus, participation in diabetes prevention programmes with a partner may improve retention, especially for ethnic minority groups.

In 2022, the US Centers for Disease Control and Prevention approved an alternative curriculum for the NDPP adapted for couples (PreventT2 Together) [58]. PreventT2 Together includes content specific to couples and encourages partners to discuss how they can best support one another to make healthy lifestyle changes [58]. Specific goals of the programme are how partners can support each other in (1) becoming more physically active; (2) eating well; (3) staying motivated to prevent type 2 diabetes, in particular when weight loss stalls; (4) managing stress; and (5) getting enough sleep [58].

In a planned first trial randomly assigning couples to PreventT2 Together or standard interventions, differences in diabetes-related health behaviours, physical and mental health and relationship functioning will be investigated (University of Utah, hospital research centre; ClinicalTrials.gov NCT05695170) [58]. Study outcomes will include physical activity, diet, body weight, waist circumference, HbA_1c_, fasting and 2 h post-load glucose, mental health (anxiety, depression) and relationship functioning (social support, relationship satisfaction, sexual intimacy) [58]. However, the results will most likely be applicable to couples in stable and supportive relationships, in which both partners want to make lifestyle changes [58].

A few other studies have evaluated the effect of couple-based interventions on specific lifestyle changes (e.g. body weight, physical activity, diet) [59, 60]. Look AHEAD was a multicentre RCT that evaluated the impact of weight loss on cardiovascular outcomes in overweight individuals with type 2 diabetes [61]. In an analysis of three study sites, untreated spouses of participants assigned to the weight loss intervention lost about 3% of their body weight, which was substantially higher than the weight loss in spouses of participants randomly assigned to usual care (0.25%) [61]. Weight change was mainly related to dietary modifications (e.g. energy intake), influenced by the shared home environment [61].

In addition, partner participation was found to influence the impact of an intervention to increase physical activity in older adults [62]. People whose partners also participated in the intervention attained higher levels of physical activity than single people and individuals whose partners did not participate [62]. It is noteworthy that the study intervention did not focus at all on partner support [62]. Nevertheless, the intervention had stronger effects on physical activity in jointly participating partners; thus, social support from partners is beneficial for physical activity. Finally, adherence to a diet intervention in people with overweight or obesity was higher when their cohabitating partners were also carrying out the same dietary changes [63].

### Methodological challenges of couple-based type 2 diabetes prevention studies

Major methodological limitations of studies on couple-based health behaviour interventions have been highlighted in a systematic review [64]. First, only a few included studies provided a theoretical background for the couple-based interventions (e.g. social support [65]). Second, couple functioning was rarely assessed and it is largely unclear whether couple-oriented type 2 diabetes prevention has a greater impact on couples with a high level of conflict related to the disease. In this respect, the level of partner support for behavioural lifestyle changes needs to be evaluated [59]. There is also probably an interaction between marital quality and preventive interactions. Study participants with a low marital quality may even experience greater benefits from couple-based interventions than couples with high marital quality [59]. In future studies, the extent to which the existence of marital problems prior to an intervention can be at least partly addressed with a couple-oriented intervention should be investigated.

Third, many studies did not report details of the intervention content. Couple-based interventions should be planned differently from those targeting individuals. Fourth, only a few studies compared couple-focused interventions with individual interventions. Ideally, three-arm studies comparing a couple-based intervention, a control group of couples and an individual intervention should be carried out [64]. This would enable any added benefits of couple-based interventions over individual interventions to be determined, as well as whether any added benefits are due to specific components of the couple-based intervention.

In another scoping review of couple-based interventions in type 2 diabetes, only four short-term (12–20 weeks) RCTs were identified, which assessed the effects of diabetes-related interventions (management of diet, exercise or stress) on both partners with diabetes and unaffected spouses [66]. Results for body weight reduction were inconclusive. Spouses in couple-based interventions had a healthier diet than spouses in individual interventions in one but not the other RCTs [66]. Overall, the evidence was limited by the low methodological quality of the included studies (e.g. inadequate and incomplete reporting of study outcomes, failed reporting of allocation concealment and blinding of study personnel) [66].

## Conclusion

There is strong evidence that cardiovascular risk factors and lifestyle factors are correlated in spouses and partners. This translates into a higher risk of type 2 diabetes in spouses and partners of people with type 2 diabetes. However, there is a lack of research on the associations between incidence of prediabetes, diabetes management and risk of diabetes complications and spousal diabetes.

Couple-oriented type 2 diabetes prevention most likely has additional benefits beyond a traditional patient-oriented approach. Couple-based studies can also help to enhance our understanding of the role of marriage and partnerships in type 2 diabetes prevention. If benefits are confirmed in future studies, healthcare providers and prevention managers should be encouraged to involve partners in type 2 diabetes prevention programmes, as this may lead to better outcomes. Ideally, well-designed RCTs with published protocols, details of allocation sequence generation and use of allocation concealment and blinding of study personnel are needed for the robust evaluation of couple-based diabetes prevention [64] and potential translation into type 2 diabetes prevention.

## Supplementary Information

Below is the link to the electronic supplementary material.

Supplementary file1 (PPTX 231 KB)

## Abbreviation

NDPP: National Diabetes Prevention Program
