# Supplementary material for: Partnership and marriage and risk of type 2 diabetes: a narrative review
Source: Diabetologia. 2025 Feb 7;68(4):704–14. doi: 10.1007/s00125-025-06360-3 (PMC11950033; doi:10.1007/s00125-025-06360-3)
Supplement: Supplementary file 1 — Supplementary file1 (PPTX 231 KB) [file 125_2025_6360_MOESM1_ESM.pptx]

## Slide 1
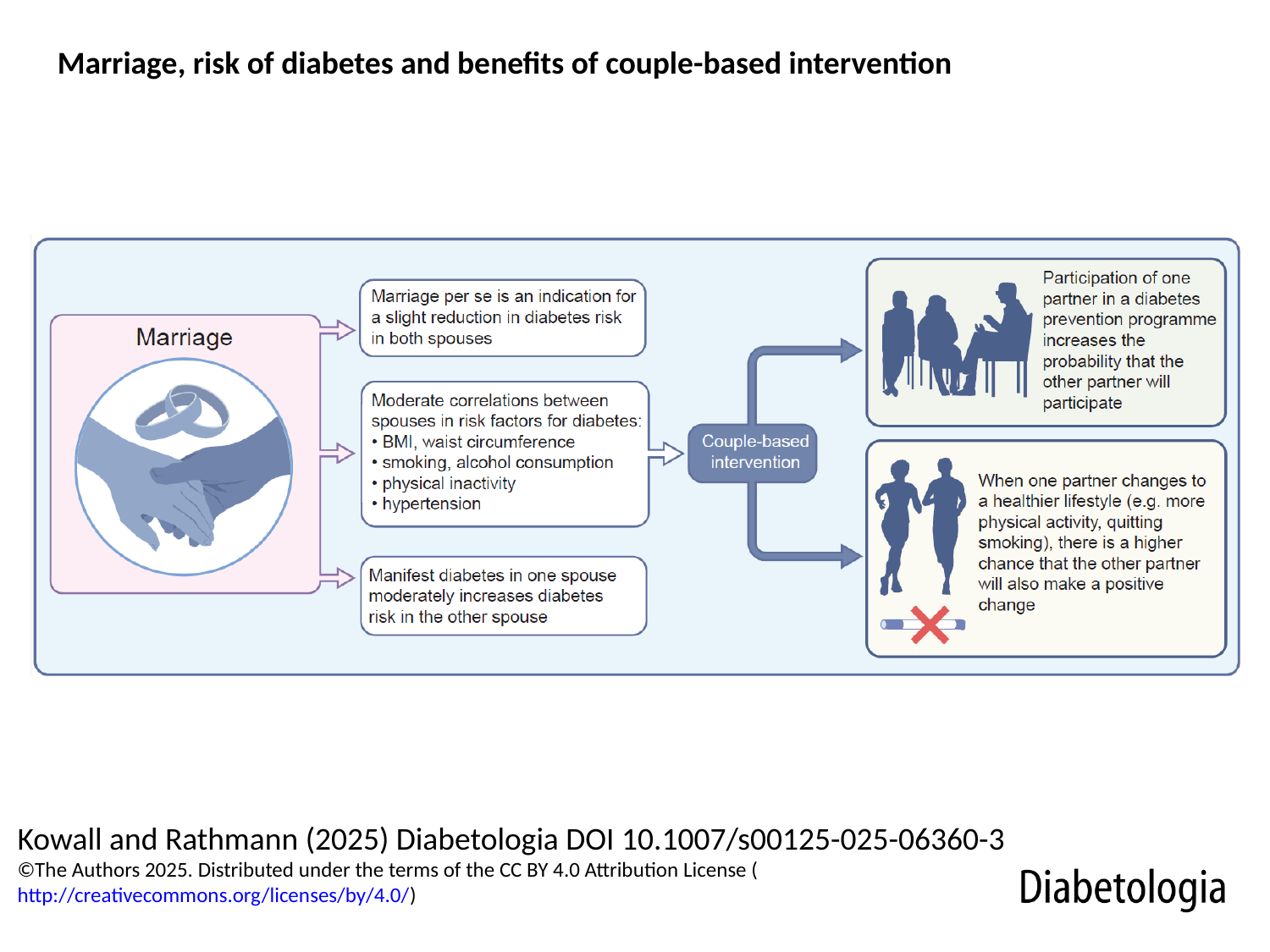

Marriage, risk of diabetes and benefits of couple-based intervention
Kowall and Rathmann (2025) Diabetologia DOI 10.1007/s00125-025-06360-3
©The Authors 2025. Distributed under the terms of the CC BY 4.0 Attribution License (http://creativecommons.org/licenses/by/4.0/)
